# Clinical efficacy of immunotherapy in combination of locoregional therapies for advanced hepatocellular carcinoma: a systematic review and meta-analysis

**DOI:** 10.3389/fimmu.2026.1706375

**Published:** 2026-02-26

**Authors:** Xinyue Chen, Mohan Huang, Ranran Liu, Lawrence Wing Chi Chan

**Affiliations:** 1 Department of Health Technology and Informatics, Hong Kong Polytechnic University, Hong Kong, Hong Kong SAR, China; 2 School of Nursing, Hong Kong Polytechnic University, Hong Kong, Hong Kong SAR, China

**Keywords:** advanced hepatocellular carcinoma (HCC), clinical efficacy, immunotherapy, locoregional therapy, meta-analysis

## Abstract

**Background:**

Hepatocellular carcinoma (HCC) is the most common type of primary liver cancer and is the leading cause of cancer-related deaths worldwide. The majority of patients with HCC are diagnosed at an advanced stage, resulting in limited treatment options. In recent years, numerous clinical trials have confirmed that immunotherapy, particularly anti-programmed cell death 1 (anti-PD-1)/programmed cell death ligand 1 (PD-L1), has emerged as a promising treatment for advanced HCC. However, in real-world practice, the clinical efficacy of adding immunotherapy to locoregional therapies remains unknown, representing a knowledge gap.

**Aims:**

This meta-analysis aims to evaluate the clinical efficacy of immunotherapy combined with locoregional therapies, including transarterial chemoembolization (TACE), hepatic artery infusion chemotherapy (HAIC), and HAIC/TACE combined with targeted agents, versus locoregional therapies alone in patients with advanced HCC.

**Methods:**

Eligible studies were identified by searching Embase, PubMed, Cochrane Library, and Web of Science. The clinical outcomes were overall survival (OS), progression-free survival (PFS), disease control rate (DCR), objective response rate (ORR), and adverse events (AEs). Pooled hazard ratios (HRs), odds ratios (ORs), and meta-regression were used to estimate clinical outcomes. Quality assessments were performed using the Newcastle–Ottawa Quality Assessment Form. The funnel plot was used for detecting publication bias.

**Results:**

Nineteen cohort studies with 3,720 patients with advanced HCC were included. The immunotherapy-added group was superior in prolonging OS [HR = 0.36, 95% confidence interval (CI) (0.29, 0.46) and *p* < 0.001], PFS [HR = 0.41, 95% CI (0.31, 0.54) and *p* < 0.001], DCR [OR = 2.17, 95% CI (1.80, 2.62), *p* < 0.001], and ORR [OR = 1.85, 95% CI (1.62, 2.12), *p* < 0.001]. The immunotherapy-added group had a higher risk of developing grade ≥3 AEs as compared to the locoregional-only therapy group [OR = 1.26, 95% CI (1.06, 1.49), *p* = 0.009]. Pooled results also indicated an increased risk of fatigue (OR = 1.17, *p* = 0.04), pneumonitis (OR = 2.97, *p* < 0.01), and myocarditis (OR = 9.08, *p* = 0.01) in the immunotherapy−added group.

**Conclusions:**

This meta-analysis compared the clinical outcomes of locoregional therapies versus immunotherapy plus locoregional therapies. This study found that adding immunotherapy was associated with improved OS, PFS, DCR, and ORR in patients with advanced HCC compared with those treated with locoregional regimens alone. Meanwhile, the addition of immunotherapy may be associated with an increased risk of grade ≥3 AEs and specific immune-related AEs in patients with advanced HCC.

**Systematic Review Registration:**

https://www.crd.york.ac.uk/PROSPERO/recorddashboard, identifier CRD420251039316.

## Introduction

Liver cancer is the sixth most common cancer worldwide and is the third leading cause of cancer-related deaths worldwide (1), accounting for approximately 700,000 deaths every year. Hepatocellular carcinoma (HCC) is the most common type of primary liver cancer, accounting for approximately 90% of cases (2). Patients with early-stage HCC may not have obvious symptoms; many patients are already in the intermediate and advanced stages when they are diagnosed. More than 70% of cases are diagnosed at the advanced stage, which means they lose the opportunity for radical treatment (3). Patients with advanced HCC experience poor prognosis, including tumor recurrence and tumor metastasis. The 5-year survival rate of patients with advanced HCC is only approximately 18% worldwide (4).

HCC treatments are determined by tumor stage and patient health status, with options including surgical resection, ablation therapy, transarterial chemoembolization (TACE), hepatic artery infusion chemotherapy (HAIC), stereotactic body radiation therapy (SBRT), targeted therapy, and immunotherapy. Local ablation, TACE, and HAIC are types of locoregional therapies (LRTs). Local ablation is the primary treatment for patients with early-stage HCC (5), while TACE and HAIC play a crucial role in managing intermediate- and advanced-stage cases. Previous studies indicated that TACE is a treatment option for unresectable cases, as it could induce tumor ischemia and necrosis by delivering drugs and targeting arterial embolization (6). Although TACE is beneficial for certain patients, its efficacy is hampered by recurrence, even in cases with initial remission (7). HAIC is an interventional treatment that directly infuses high concentrations of chemotherapy drugs into liver tumors through the hepatic artery, resulting in significant local antitumor effects (8). Although the Japan Society of Hepatology (JSH) has approved HAIC as a feasible option for treating advanced HCC, there remains international controversy regarding the clinical efficacy of HAIC (9).

Approximately 50%–60% of patients with advanced HCC choose systemic therapy (10), and majority of unresectable patients would undergo targeted therapies and immunotherapy. In recent years, because of significant advancements in targeted therapy, many agents have been approved as first-line therapy for HCC. According to the results of phase III clinical trials known as SHARP and the Asia-Pacific trial for patients with advanced HCC, patients who were treated with sorafenib had better survival outcomes compared to the placebo group (11). For decades, sorafenib has been the first-line therapy for patients with advanced HCC. Nevertheless, sorafenib demonstrates limited antitumor activity, and the acquired resistance to sorafenib decreases its therapeutic benefits (12). In 2019, a large-scale phase III clinical trial known as IMbrave150 indicated that atezolizumab (PD-L1 inhibitor)/bevacizumab (VEGF antibody) improved overall survival (OS) and progression-free survival (PFS) outcomes compared to the sorafenib group (13). The approval of the atezolizumab combined with bevacizumab therapy by the Food and Drug Administration (FDA) has established it as one of the first-line treatments for advanced HCC. The advent of immunotherapy, particularly anti-programmed cell death 1 (anti-PD-1)/programmed cell death ligand 1 (PD-L1), has changed the treatment landscape of patients with advanced HCC. One clinical trial found that TACE plus lenvatinib plus pembrolizumab could bring significant and meaningful improvement in PFS in patients with HCC (14). One multiregional phase III study indicated that durvalumab plus bevacizumab plus TACE has potential to become a new standard treatment for patients with unresectable HCC (15). Another single-arm clinical trial demonstrated that sintilimab plus lenvatinib and TACE/HAIC showed promise in patients with unresectable HCC (16). In clinical trials, adding immunotherapy drugs to HAIC/TACE or HAIC/TACE combined with targeted agents has been shown to be effective. Therefore, incorporating immunotherapy into LRTs in real-world clinical practice to improve survival outcomes for patients with advanced HCC is a highly significant and worthy consideration.

This meta-analysis aims to evaluate the clinical efficacy of immunotherapy (targeting PD-1/PD-L1) combined with LRTs, including TACE, HAIC, and HAIC/TACE combined with targeted agents, versus LRTs alone in patients with advanced HCC. Our study seeks to comprehensively assess multiple outcomes, including OS, PFS, disease control rate (DCR), and objective response rate (ORR). This meta-analysis specifically incorporated cohort studies to provide insights relevant to clinical practice and strengthen the generalizability of findings in real-world settings.

## Method

### Literature search

A relevant article search was performed on four databases: Embase, PubMed, Cochrane Library, and Web of Science from database inception to April 2025. Search items included “Advanced hepatocellular carcinoma”, “Unresectable hepatocellular carcinoma”, “Immunotherapy”, “Transarterial chemoembolization”, “Hepatic artery infusion chemotherapy”, “PD-1 inhibitors”, and “PD-L1 inhibitors”. In addition to databases, we supplemented our search by manually screening reference lists of relevant reviews to identify additional eligible studies.

### Literature selection

The cohort studies were included based on the following inclusion criteria: (1) patients age ≥18 years; (2) BCLC B or C stage; (3) patients with Child–Pugh A or B; (4) no prior related treatment for HCC; (5) only English-language publications; (6) patients treated with LRTs/immunotherapy + LRTs; and (7) reporting clinical outcomes. Literature selection was independently performed by two reviewers.

Traditional therapies include TACE, HAIC, and any combination of the TACE/HAIC with targeted agents.

### Data extraction and quality assessment

Data extraction was independently performed by two reviewers. The following types of information were extracted:

1. Study identification: Journal name, publication date, and ethics approval status.
2. Patient characteristics: Sample size, treatment regimen, age, gender, Eastern Cooperative Oncology Group (ECOG) performance status score, Barcelona Clinic Liver Cancer (BCLC) stage, Child–Pugh liver function class, and alpha-fetoprotein (AFP) serum concentration levels.
3. Intervention: Agents used in immunotherapy treatment regimens.
4. Comparison: Agents used in locoregional treatment regimens.
5. Outcomes: OS, PFS, DCR, ORR, and adverse events (AEs).

Quality assessment was performed based on the Newcastle–Ottawa Quality Assessment Form for cohort studies (17).

### Statistical analysis

Before meta-analysis, heterogeneity assessment was performed by using *I*^2^ statistics. The fixed-effects model would be chosen if *I*^2^ ≤ 50%, and the random-effects model would be chosen if *I*^2^ > 50%. The generic inverse variance method was used for time-to-event outcomes, hazard ratio (HR) and 95% confidence interval (CI) were directly obtained from study results, and the standard error can be calculated based on the 95% CI. The Mantel–Haenszel method was used for dichotomous outcomes, effects would be expressed as odds ratios (ORs) with 95% CI. The funnel plot was used for detecting publication bias. To further investigate the potential sources of high heterogeneity, meta-regression analyses were performed. Statistical analysis was performed by RevMan version 5.3 and R version 4.3.1.

## Results

### Search process and included studies

**Figure 1:**
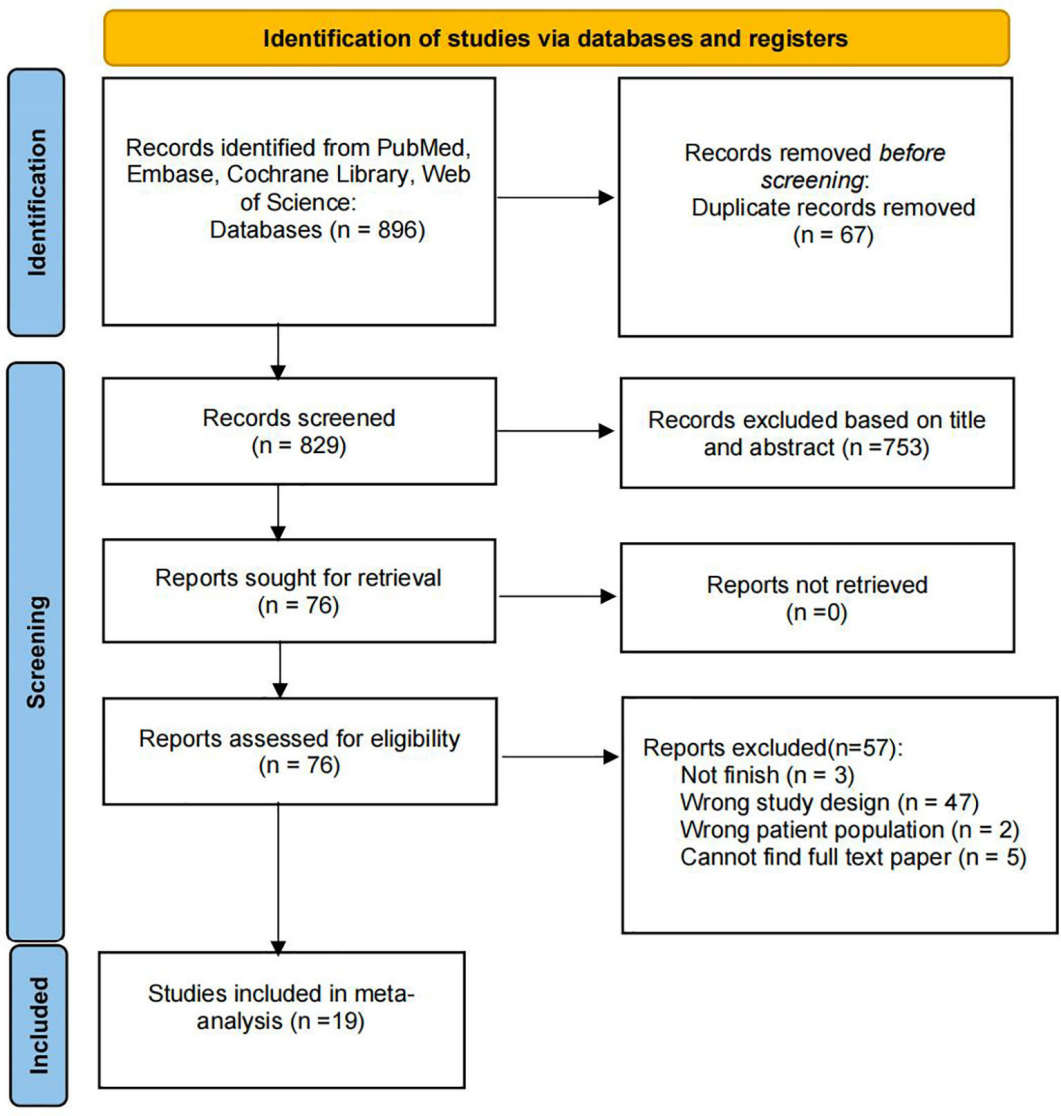
Flowchart of literature search and selection.

A total of 896 records were initially identified from PubMed, Embase, Cochrane Library, and Web of Science. After deleting 67 duplicate records, we screened the full-text paper according to the inclusion criteria. Finally, 19 eligible studies were included in the meta-analysis (18–36). The article searches and selection process are shown in **Figure 1**.

### Description of baseline characteristics

All 19 included studies were published after 2021. A total of 3,720 patients with advanced HCC were included in this meta-analysis. All 19 studies were retrospective cohort studies, which were conducted in China in the past 10 years. Nineteen studies only included BCLC B/C stage patients who had the Child–Pugh liver function of A/B. The immunotherapy used in the cohort studies included PD-1 inhibitors and PD-L1 inhibitors. Characteristics of the included studies are shown in **Table 1**. According to the Newcastle–Ottawa Quality Assessment Form for cohort studies, all studies demonstrated high quality, with scores ranging from eight to nine stars (**Table 2**).

**Table 1:** Characteristics of included studies.

Study ID	Publication year	Study design	Treatment regimen	Details	Total sample size	Study period	Population: age range	ECOG performance status score	BCLC stage	Child–Pugh liver function	Male (%)	AFP >400 (%)
Cai 2022 (18)	2022	Retrospective cohort study	TACE+targeted therapy+immunotherapy/TACE+targeted therapy	TACE+lenvatinib+PD-1 inhibitor/TACE+lenvatinib	81	Jan 2019 to Dec 2020	18 to 75	0–1	C	A/B	90.2	51.2
Chen 2022 (19)	2021	Retrospective cohort study	TACE+targeted therapy+immunotherapy/TACE+targeted therapy	TACE+lenvatinib+pembrolizumab/TACE+lenvatinib	142	Jul 2016 to Jul 2020	35 to 69	0–1	B/C	A	52.9	64.3
Xia 2022 (30)	2022	Retrospective cohort study	TACE+targeted therapy+immunotherapy/TACE+targeted therapy	TACE+apatinib+PD-1 inhibitors/TACE+apatinib	118	Dec 2018 to Jun 2021	18 to 75	0–1	C	A/B	91.2	38.2
Li 2024 (24)	2023	Retrospective cohort study	HAIC+immunotherapy/HAIC	HAIC+PD-1 inhibitors/HAIC	442	Mar 2018 to Dec 2019	18 to 80	0–1	C	A/B	87.3	58.8
Yang 2023 (32)	2023	Retrospective cohort study	TACE+targeted therapy+immunotherapy/TACE+targeted therapy	TACE+lenvatinib+PD-(L)1 inhibitors/TACE+lenvatinib	122	2019 to 2022	18 to 75	0–1	B/C	A/B	79.7	51.6
Wu 2024 (29)	2024	Retrospective cohort study	TACE+targeted therapy+immunotherapy/TACE+targeted therapy	TACE+apatinib+ICI/TACE+apatinib	90	May 2020 to Jul 2023	≥18	0–1	C	A/B	89.5	NR
Wu 2025 (28)	2025	Retrospective cohort study	TACE+targeted therapy+immunotherapy/TACE+targeted therapy	TACE+lenvatinib+sintilimab/TACE+lenvatinib	57	Sept 2019 to Sept 2022	37 to 80	0–1	B/C	A/B	90	NR
Jiang 2024 (23)	2024	Retrospective cohort study	TACE+targeted therapy+immunotherapy/TACE+targeted therapy	TACE+lenvatinib+tislelizumab/TACE+lenvatinib	136	Jan 2021 to Jun 2023	18 to 75	0–1	B/C	A/B	86.8	35.3
Zhao 2024 (33)	2024	Retrospective cohort study	TACE+targeted therapy+immunotherapy/TACE+targeted therapy	TACE+lenvatinib+tislelizumab/TACE+lenvatinib	169	Mar 2021 to Sept 2023	NR	0–1	B/C	A/B	75.7	54.4
Chen 2024 (5)	2024	Retrospective cohort study	TACE+HAIC+targeted therapy+immunotherapy/TACE+HAIC+targeted therapy	TACE+HAIC+TKIs+PD-1/TACE+HAIC+TKIs	78	Nov 2020 to Feb 2024	18 to 80	0–2	B/C	A/B	56.5	NR
Xiang 2023 (31)	2023	Retrospective cohort study	TACE+targeted therapy+immunotherapy/TACE+targeted therapy	TACE+lenvatinib+camrelizumab/TACE+lenvatinib	82	Nov 2018 to Jun 2021	NR	0–1	B/C	A/B	84.8	57.6
Wang 2025 (27)	2025	Retrospective cohort study	TACE+targeted therapy+immunotherapy/TACE+targeted therapy	TACE+TKIs+PD-1 inhibitors/TACE+TKIs	174	Dec 2018 to Jan 2023	18 to 75	0–1	B/C	A/B	55.7	27
Sun 2024 (26)	2024	Retrospective cohort study	TACE+targeted therapy+immunotherapy/TACE+targeted therapy	TACE+sorafenib+camrelizumab/TACE+sorafenib	78	Jan 2018 to Dec 2021	NR	0–1	B/C	A/B	88.5	46.2
Mei 2021 (25)	2021	Retrospective cohort study	HAIC+immunotherapy/HAIC	HAIC+PD-1 inhibitors/HAIC	229	Nov 2018 to Dec 2019	18 to 75	NR	B/C	A	91%	56
Duan 2023 (21)	2023	Retrospective cohort study	TACE+targeted therapy+immunotherapy/TACE+targeted therapy	TACE+apatinib+camrelizumab/TACE+apatinib	960	Jan 2019 to Jun 2021	18 to 80	0–1	B/C	A/B	82.6	59
Guo 2022 (22)	2022	Retrospective cohort study	TACE+targeted therapy+immunotherapy/TACE+targeted therapy	TACE+lenvatinib+PD-1 inhibitors/TACE+lenvatinib	96	Jan 2018 to Jan 2022	≥18	0–1	B/C	A	86.7	68
Li 2023 (35)	2023	Retrospective cohort study	TACE+target therapy+immunotherapy/TACE+target therapy	TACE+donafenib+PD-1 inhibitors/TACE+donafenib	323	July 2021 to July 2022	18 to 80	0–2	B/C	A/B	83.3	61.3
Zhu 2022 (34)	2022	Retrospective cohort study	TACE+target therapy+immunotherapy/TACE+target therapy	TACE+apatinib+camrelizumab/TACE+apatinib	102	Jan 2018 to Jan 2022	NR	0–1	B/C	A/B	85.3	NR
Sheng 2024 (36)	2024	Retrospective cohort study	TACE+target therapy+immunotherapy/TACE+target therapy	TACE+lenvatinib+PD-1 inhibitors/TACE+lenvatinib	241	Jan 2016 to Dec 2021	NR	0–2	B/C	A/B	84.2	32.4

**Table 2:** Newcastle–Ottawa scale for cohort studies.

Study ID	Selection				Comparability		Outcome			Total scores
	Representativeness	Selection of non-exposure	Ascertainment of exposure	Outcome not present at start	Comparability on most important factors	Comparability on other risk factors	Assessment of outcome	Adequate follow-up time	Complete follow-up	
Cai 2022 (18)	✓	✓	✓	✓	✓	✓	✓	✓	✓	9
Chen 2022 (19)	✓	✓	✓	✓	✓	✓	✓	✓	✓	9
Xia 2022 (30)	✓	✓	✓	✓	✓	✓	✓	✓	✓	9
Li 2024 (24)	✓	✓	✓	✓	✓	✓	✓	✓	✓	9
Yang 2023 (32)	✓	✓	✓	✓	✓	✓	✓	×	✓	8
Wu 2024 (29)	✓	✓	✓	✓	✓	✓	✓	✓	✓	9
Wu 2025 (28)	✓	✓	✓	✓	✓	✓	✓	✓	✓	9
Jiang 2024 (23)	✓	✓	✓	✓	✓	✓	✓	×	✓	8
Zhao 2024 (33)	✓	✓	✓	✓	✓	✓	✓	✓	✓	9
Chen 2024 (5)	✓	✓	✓	✓	✓	✓	✓	✓	✓	9
Xiang 2023 (31)	✓	✓	✓	✓	✓	✓	✓	✓	✓	9
Wang 2025 (27)	✓	✓	✓	✓	✓	✓	✓	✓	✓	9
Sun 2024 (26)	✓	✓	✓	✓	✓	✓	✓	✓	✓	9
Mei 2021 (25)	✓	✓	✓	✓	✓	✓	✓	✓	✓	9
Duan 2023 (21)	✓	✓	✓	✓	✓	✓	✓	✓	✓	9
Guo 2022 (22)	✓	✓	✓	✓	✓	✓	✓	✓	✓	9
Li 2023 (35)	✓	✓	✓	✓	✓	✓	✓	✓	✓	9
Zhu 2022 (34)	✓	✓	✓	✓	✓	✓	✓	✓	✓	9
Sheng 2024 (36)	✓	✓	✓	✓	✓	✓	✓	✓	✓	9

### Overall survival

**Figure 2:**
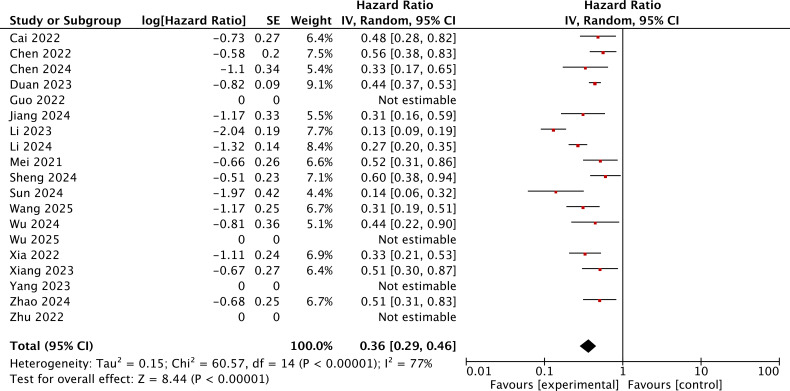
The forest plot for OS of the immunotherapy-added group and the traditional therapy group and locoregional therapy group.

A total of 15 studies reported the HR for OS of the immunotherapy-added group and the locoregional-only therapy group. The heterogeneity was assessed using the *I*^2^ statistic and *p*-value. *I*^2^ = 77% (>50%) indicated that the random-effects model should be chosen. The pooled HR for OS was 0.36, with 95% CI of (0.29, 0.46) and *p* < 0.001. This result indicated that the immunotherapy-added group was significantly associated with improved OS as compared to the locoregional-only therapy group (**Figure 2**).

### Progression-free survival

**Figure 3:**
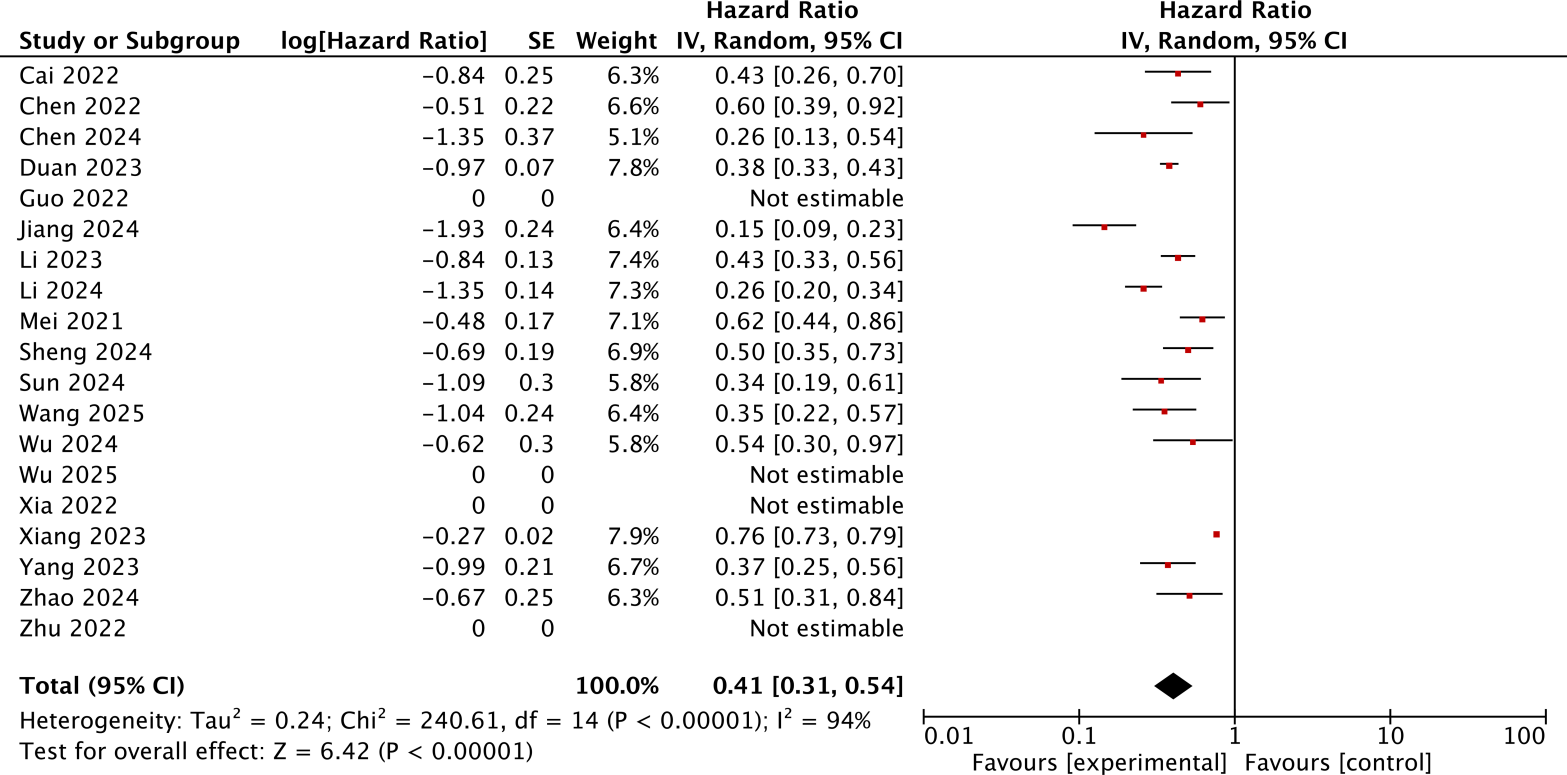
The forest plot for PFS of the immunotherapy-added group and the traditional therapy group and locoregional therapy group.

A total of 15 studies reported the HR for PFS of the immunotherapy-added group and the locoregional-only therapy group. The heterogeneity was assessed using the *I*^2^ statistic and *p*-value. *I*^2^ = 94% (>50%) indicated that the random-effects model should be chosen. The pooled HR for PFS was 0.41, with a 95% CI of (0.31, 0.54) and *p* < 0.001. This result indicated that the immunotherapy-added group was significantly associated with improved PFS as compared to the locoregional-only therapy group (**Figure 3**).

### Tumor response

A total of 18 studies reported the tumor response of the immunotherapy-added group and the traditional therapy group, including complete response, partial response, progressive disease, stable disease, DCR, and ORR.

**Figure 4:**
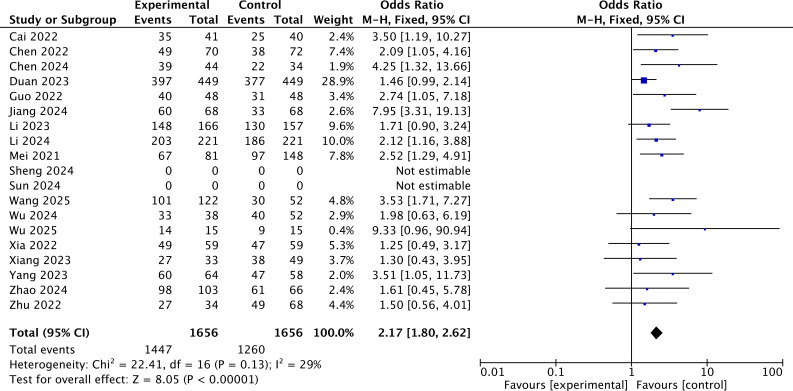
The forest plot for DCR of the immunotherapy-added group and the traditional therapy group and locoregional therapy group.

According to the results of the DCR, the heterogeneity was assessed using the *I*^2^ statistic and *p*-value. *I*^2^ = 29% (<50%) indicated that the fixed-effects model should be chosen. The pooled OR demonstrated that added immunotherapy significantly increased the DCR compared to locoregional-only therapy [OR = 2.17, 95% CI (1.80, 2.62), *p* < 0.001] (**Figure 4**).

**Figure 5:**
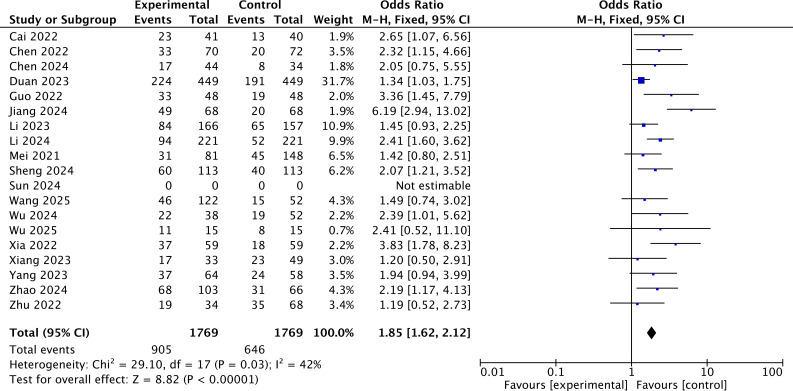
The forest plot for ORR of the immunotherapy-added group and the traditional therapy group and locoregional therapy group.

According to the results of the ORR, the heterogeneity was assessed using the *I*^2^ statistic and *p*-value. *I*^2^ = 42% (<50%) indicated that the fixed-effects model should be chosen. The pooled OR demonstrated that added immunotherapy significantly increased the ORR compared to locoregional-only therapy [OR = 1.85, 95% CI (1.62, 2.12), *p* < 0.001] (**Figure 5**).

### Safety analysis

**Figure 6:**
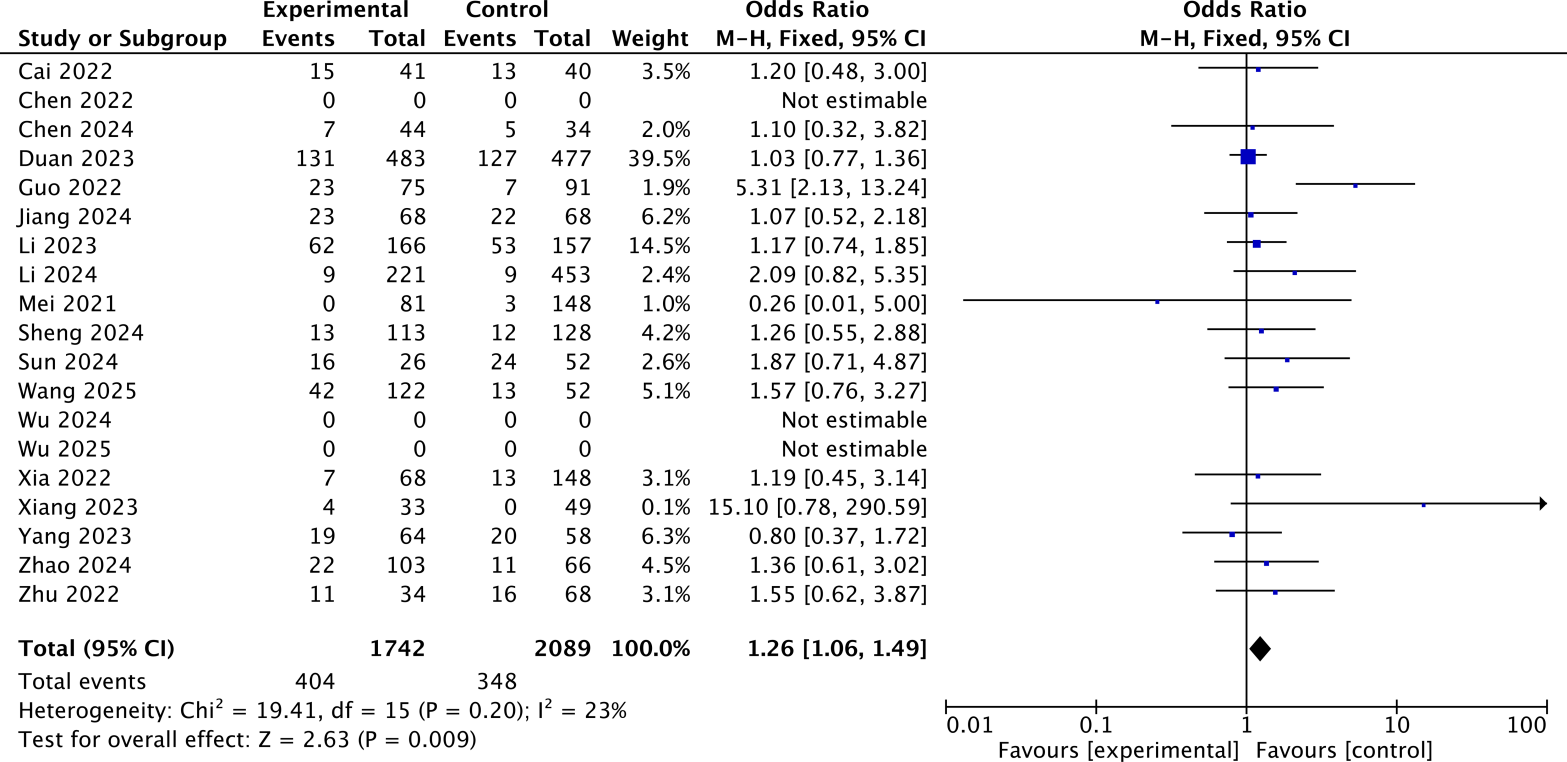
The forest plot for grade ≥3 AEs.

Safety was firstly assessed by evaluating the incidence of grade 3 or higher AEs across 16 studies. The rate of grade ≥3 AEs was reported in a total of 16 studies. According to the results of the grade ≥3 AEs, heterogeneity was assessed using the *I*^2^ statistic and *p*-value. *I*^2^ = 23% (<50%) indicated that the fixed-effects model should be chosen. The pooled OR demonstrated that the immunotherapy-added group showed a higher probability of experiencing grade ≥3 AEs as compared to the locoregional-only group [OR = 1.26, 95% CI (1.06, 1.49), *p* = 0.009] (**Figure 6**).

**Figure 7:**
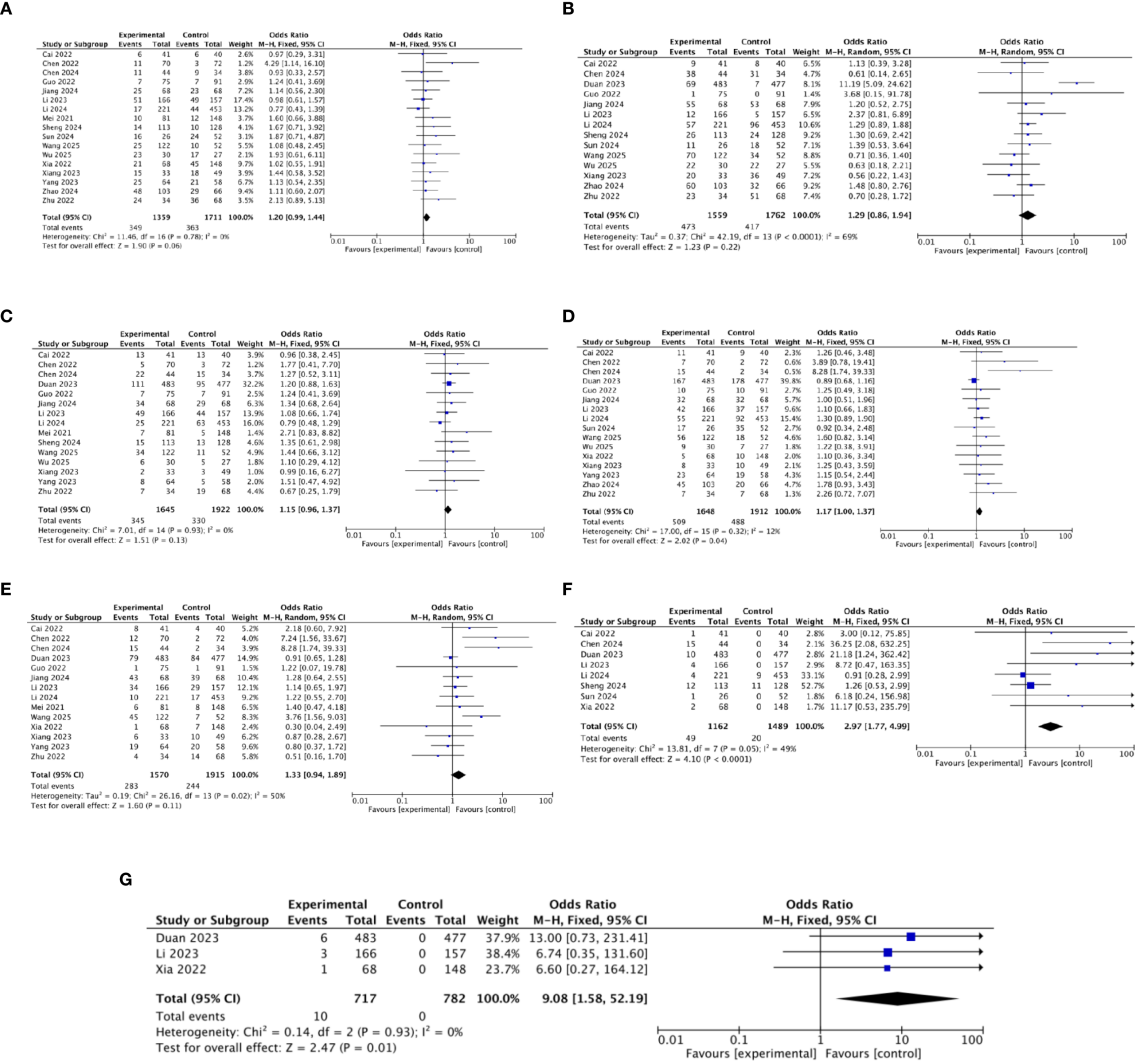
**(a)** The forest plot for nausea/vomiting. **(b)** The forest plot for abdominal pain. **(c)** The forest plot for diarrhea. **(d)** The forest plot for fatigue. **(e)** The forest plot for rash. **(f)** The forest plot for pneumonitis. **(g)** The forest plot for myocarditis.

Safety was further assessed by examining the incidence of immune-related AEs (irAEs) of any grade across the included studies. IrAEs affect multiple organ systems (37), with skin and gastrointestinal irAEs being the most common (38). This meta-analysis focused on the following irAEs: nausea/vomiting (38), abdominal pain (39), diarrhea (40), fatigue (41), rash (42), pneumonitis (43), and myocarditis (44) (**Figures 7a–g**). Among these, pneumonitis and myocarditis represent rare but serious events requiring clinical attention. The pooled OR demonstrated that the immunotherapy-added group showed a higher probability of experiencing fatigue (OR = 1.17, *p* = 0.04), pneumonitis (OR = 2.97, *p* < 0.01), and myocarditis (OR = 9.08, *p* = 0.01) as compared to the locoregional-only group. The pooled OR showed no significant difference in experiencing nausea/vomiting, abdominal pain, diarrhea, and rash between different regimen groups.

### Publication bias

**Figure 8:**
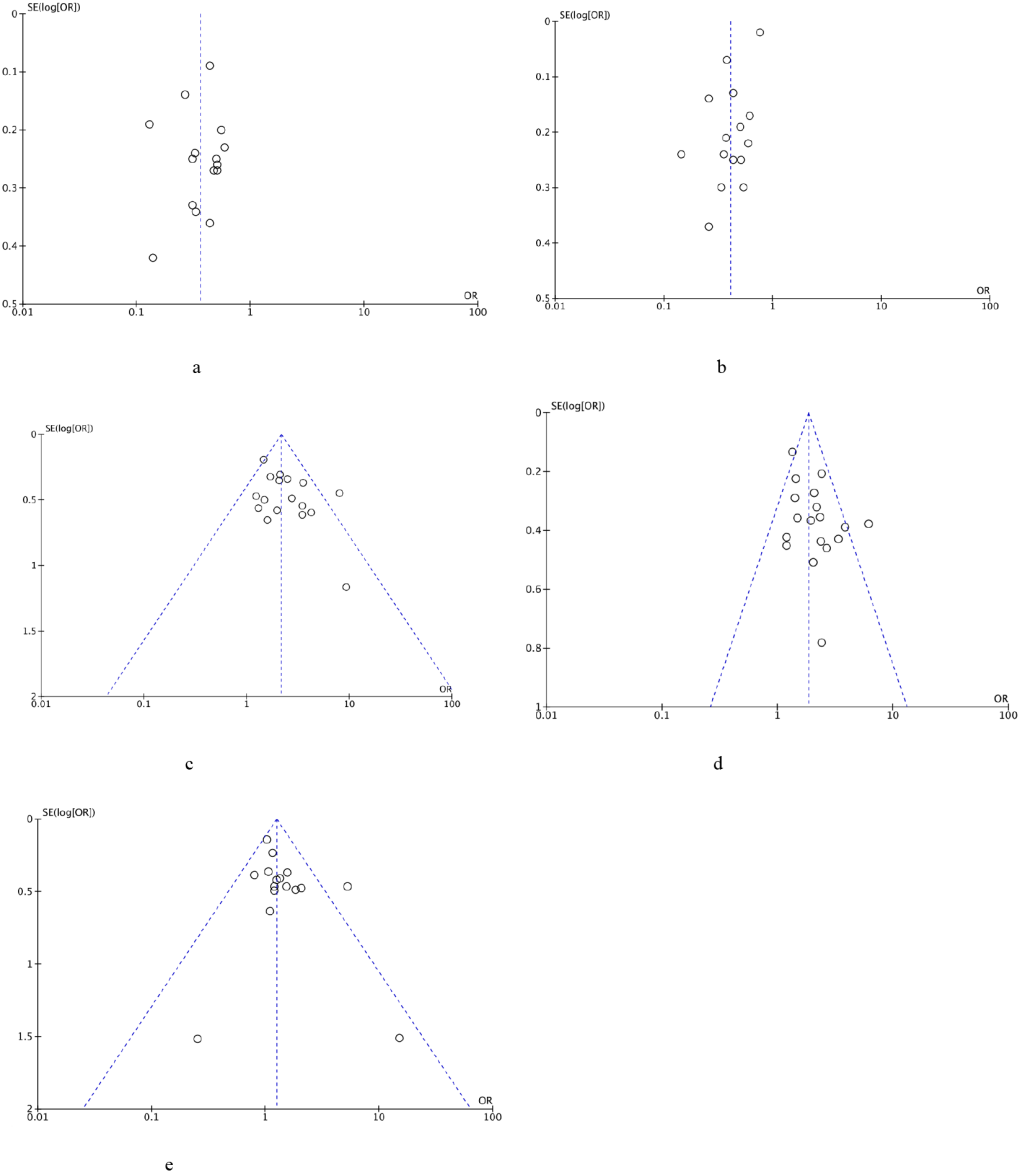
**(a)** Funnel plot of OS. **(b)** Funnel plot of PFS. **(c)** Funnel plot of DCR. **(d)** Funnel plot of ORR. **(e)** Funnel plot of grade ≥3 AEs.

This meta-analysis used the funnel plot to detect the potential publication bias for OS, PFS, DCR, ORR, and grade ≥3 AEs (**Figures 8a–e**). All funnel plots were approximately symmetrical and showed no substantial evidence of publication bias.

### Subgroup analysis

#### Subgroup analysis of OS, PFS, DCR, and ORR for immunotherapy with TACE and TKIs

**Figure 9:**
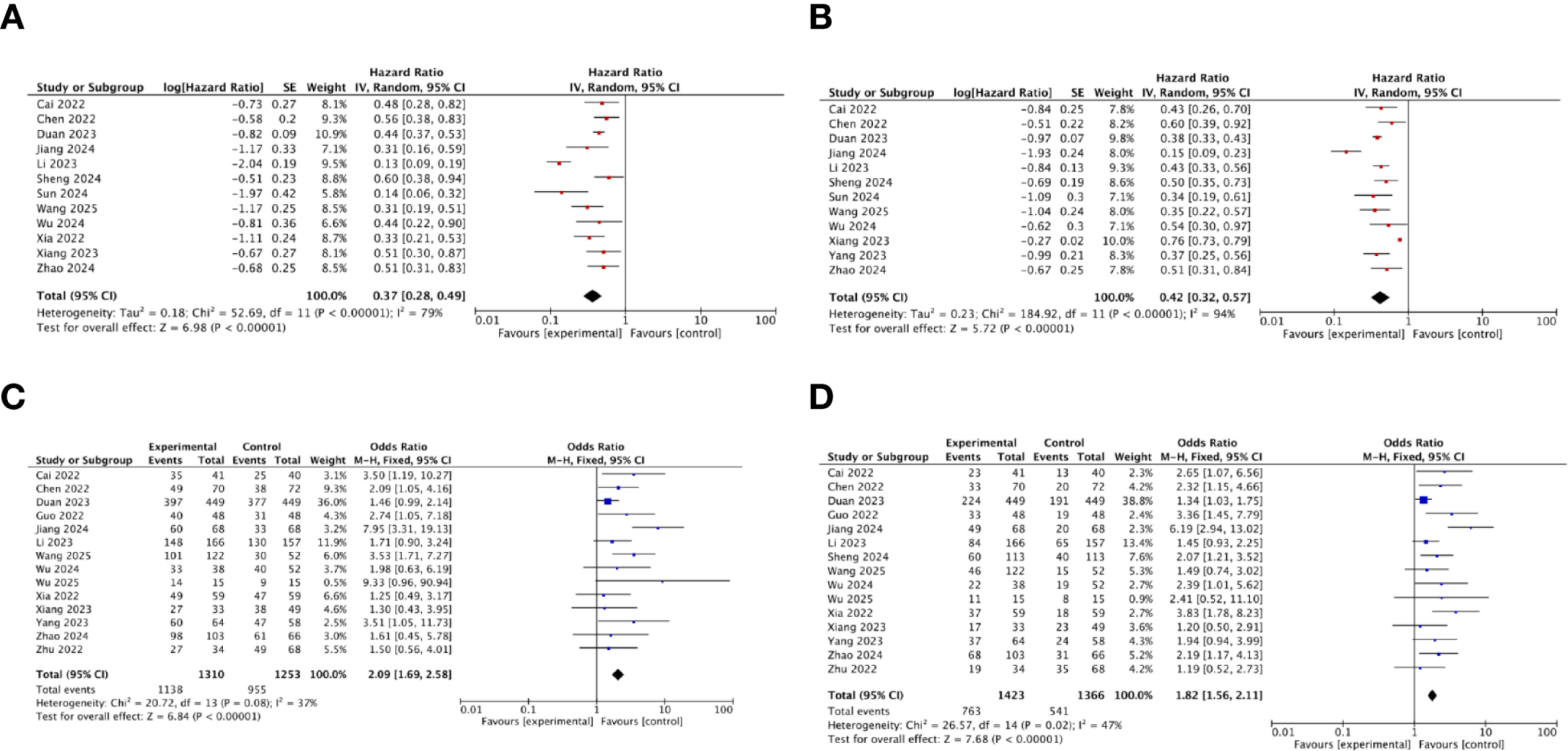
**(a)** Subgroup analysis of OS for immunotherapy–TACE–TKIs. **(b)** Subgroup analysis of PFS for immunotherapy–TACE–TKIs. **(c)** Subgroup analysis of DCR for immunotherapy–TACE–TKIs. **(d)** Subgroup analysis of ORR for immunotherapy–TACE–TKIs.

Various locoregional treatment regimens were reported in the included studies, among which 16 investigated the triple−combination therapy of TACE, TKIs, and immunotherapy. Subgroup analysis regarding OS, PFS, DCR, and ORR for this triple combination therapy was performed (**Figures 9a–d**). The pooled HR for OS and PFS was 0.37 and 0.42, respectively. The result also indicated that the immunotherapy–TACE–TKIs therapy group was significantly associated with improved OS and PFS as compared to the TACE–TKIs regimen. The pooled OR for DCR and ORR was 2.09 and 1.82, respectively, suggesting that the addition of immunotherapy significantly increased the DCR and ORR as compared to TACE–TKIs group.

#### Subgroup analysis of OS, PFS, DCR, and ORR for immunotherapy combined with HAIC

**Figure 10:**
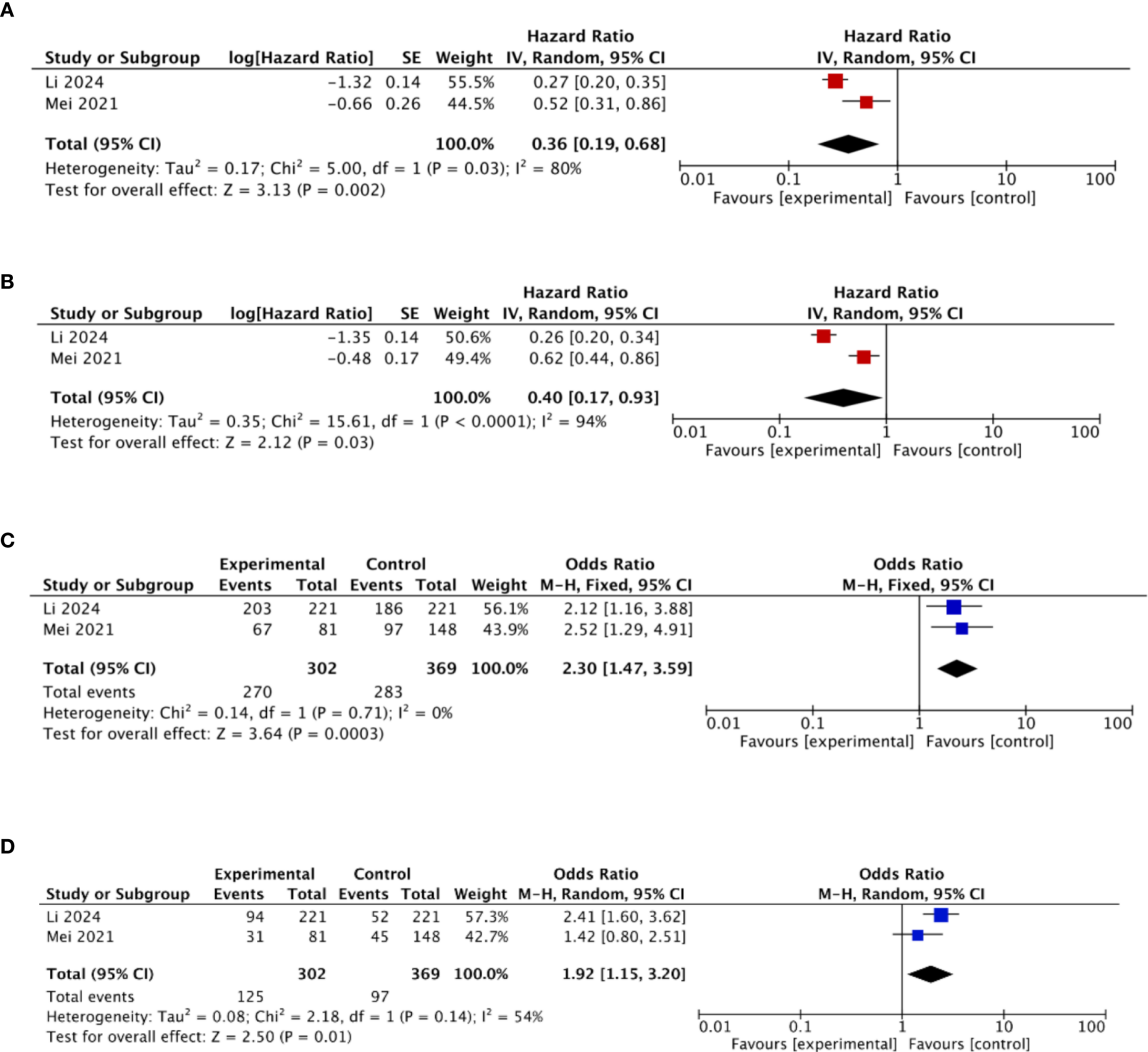
**(a)** Subgroup analysis of OS for immunotherapy–HAIC. **(b)** Subgroup analysis of PFS for immunotherapy–HAIC. **(c)** Subgroup analysis of DCR for immunotherapy–HAIC. **(d)** Subgroup analysis of ORR for immunotherapy–HAIC.

Two studies investigated the combination of HAIC and immunotherapy. Subgroup analysis of OS, PFS, DCR, and ORR for immunotherapy plus HAIC was conducted (**Figures 10a–d**). The pooled HRs for OS and PFS were 0.36 and 0.40, respectively, indicating significantly improved survival outcomes compared with HAIC alone. The pooled ORs for DCR and ORR were 2.30 and 1.92, respectively, suggesting that adding immunotherapy markedly enhanced DCR and ORR compared to HAIC monotherapy.

### Exploration of heterogeneity by meta-regression

Heterogeneity for PFS and OS was high. This study included covariates related to treatment effect and population/study design to perform the meta-regression.

Meta-regression showed that studies including pretreated (non-first line) patients tended to report a more pronounced PFS benefit with immunotherapy plus LRT compared with first-line cohort studies (*p* = 0.01). Meanwhile, studies with higher proportions of patients with AFP > 400 showed a borderline significant trend toward elevated HRs for PFS (*p* = 0.05). These findings suggest that treatment line and the proportion of patients with elevated AFP may be important sources of heterogeneity (**Supplementary Table 1**; **Supplementary Figures 1**, **S2**). Using the same set of covariates in meta-regression for OS did not identify any statistically significant associations (**Supplementary Table 2**).

## Discussion

HCC is the most common type of primary liver cancer and poses a huge health burden worldwide. A single treatment regimen is not sufficient, and a combination of multiple therapies is common and essential for patients with advanced HCC in recent years. One open-label, multicenter randomized trial (NCT01217034) found that TACE plus sorafenib would improve PFS for patients (45). Another randomized controlled phase II clinical study (NCT04066543) found that anlotinib combined with TACE was a safe and effective regimen for patients with intermediate and advanced-stage HCC (46). One larger multicenter clinical trial (NCT02774187) reported that sorafenib plus HAIC of oxaliplatin, fluorouracil, and leucovorin (FOLFOX) could improve OS for patients with HCC with portal vein invasion (47).

Immunotherapy has emerged as a promising and revolutionary therapy for HCC and has achieved major development in recent years. One non-randomized phase II trial (NCT02702414) found that pembrolizumab (PD-1 inhibitor) had clinical efficacy in patients with advanced HCC who had previously been treated with sorafenib (48). Another clinical trial (NCT01693562) reported that durvalumab (PD-L1 inhibitor) showed promising antitumor activity and improvement in OS in patients (49).

Multiple clinical trials worldwide have confirmed that immunotherapy combined with LRTs demonstrates favorable clinical efficacy and acceptable toxicities in patients with advanced HCC. A phase I/II study (NCT02519348) indicated that the durvalumab/tremelimumab combination had no unexpected safety signals, and phase III is ongoing (50). A multiregional phase III study (NCT03778957) had found that a combination of durvalumab, bevacizumab, and TACE could significantly improve PFS in patients with HCC and had promising clinical outcomes (51).

This meta-analysis enrolled 19 cohort studies with a total of 3,720 patients, all of which demonstrated high quality based on the Newcastle–Ottawa Quality Assessment Form for cohort studies. This study aimed to evaluate whether the addition of PD-1/PD-L1 inhibitors to LRTs (TACE, HAIC, or HAIC/TACE combined with targeted agents) could lead to better clinical outcomes for patients with advanced HCC in real-world settings. Therefore, this meta-analysis systematically compares patients receiving LRTs alone versus those receiving combined immunotherapy and locoregional regimens.

To date, this is the first meta-analysis study comparing the clinical outcomes of LRTs versus immunotherapy plus locoregional regimens. This study found that adding immunotherapy was associated with improved OS, PFS, DCR, and ORR in patients with advanced HCC compared with those treated with locoregional regimens alone. As all studies included in this meta-analysis are retrospective, it is important to contextualize our findings with evidence from phase III randomized controlled trials. Preliminary results from these trials have suggested a trend toward improved clinical outcomes with combined immunotherapy and locoregional treatments, which is consistent with our pooled findings. LEAP-012 (52) demonstrated that TACE plus lenvatinib plus pembrolizumab significantly improved PFS compared with TACE plus placebo in patients with unresectable, non-metastatic HCC. Our subgroup analysis of the triple−combination therapy of TACE, TKIs, and immunotherapy yielded consistent findings: the immunotherapy–TACE–TKIs combination was significantly associated with improved PFS compared to the TACE–TKIs regimen alone. The EMERALD-1 trial (53) demonstrated that TACE combined with durvalumab plus bevacizumab significantly improved PFS in patients with HCC. Although our meta-analysis did not include studies evaluating the triple−combination therapy of TACE, PD-L1, and anti-VEGF combination, the findings from this clinical trial provide valuable direction for future meta-analyses investigating this specific regimen. Another trial (54) demonstrated that HAIC combined with lenvatinib and PD-1 inhibitors yielded superior clinical efficacy in patients with advanced HCC with portal vein tumor thrombosis. Our study specifically examined HAIC and PD-1 immunotherapy, and our subgroup analysis revealed consistent findings: adding immunotherapy to HAIC significantly improved OS, PFS, DCR, and ORR compared with HAIC monotherapy alone.

The choice of locoregional treatment regimen may considerably impact the clinical outcomes of patients with HCC. TACE blocks the arterial blood supply to HCC, resulting in tumor ischemia and necrosis, whereas HAIC delivers chemotherapeutic agents directly into the tumor-feeding artery, increasing the local drug concentration within the liver and enhancing the antitumor efficacy (55). Given these differences, we conducted subgroup analyses of OS, PFS, DCR, and ORR for immunotherapy combined with different locoregional regimens. The results showed that both the immunotherapy–TACE–TKIs and immunotherapy–HAIC combinations were associated with significant improvements in survival and tumor response outcomes compared with their respective locoregional regimens alone.

Regarding the safety, our meta-analysis indicated that there was a higher risk of developing grade ≥3 AEs in the immunotherapy-added group, and this result was consistent with previous studies (56, 57). In particular, irAEs associated with the addition of immune checkpoint inhibitors should be carefully considered. The pooled results demonstrated that the immunotherapy-added group showed a higher probability of experiencing fatigue, pneumonitis, and myocarditis as compared to the locoregional-only group. Therefore, close monitoring and clinical attention are crucial to balance therapeutic efficacy and safety in patients with advanced HCC.

This meta-analysis has several limitations. Firstly, all included studies were retrospective in nature. The intrinsic variability among these studies in terms of patient baseline characteristics, disease stages, and treatment protocols may have contributed to the high heterogeneity observed for PFS and OS. The meta-regression analysis was performed to explore potential sources of heterogeneity, and the results suggested that treatment line and the proportion of patients with elevated AFP levels might be important contributing factors. However, because of unmeasured confounders inherent to retrospective cohorts and inconsistencies in data reporting across studies, many other potential variables could not be included in the meta-regression because of data limitations. Secondly, all the included studies were cohort studies conducted in Chinese populations. Therefore, future research should incorporate data from other ethnic groups to enhance the generalizability of the findings.

In conclusion, this meta-analysis demonstrated that adding immunotherapy to locoregional regimens was associated with enhanced OS, PFS, DCR, and ORR in patients with advanced or unresectable HCC. Nonetheless, close attention should be paid to the higher incidence of irAEs and grade ≥3 AEs observed in the immunotherapy-added groups.

## Data Availability

The original contributions presented in the study are included in the article/**Supplementary Material**. Further inquiries can be directed to the corresponding author.
