# Supplementary material for: Clinical efficacy of immunotherapy in combination of locoregional therapies for advanced hepatocellular carcinoma: a systematic review and meta-analysis
Source: Front Immunol. 2026 Feb 26;17:1706375. doi: 10.3389/fimmu.2026.1706375 (PMC12979554; doi:10.3389/fimmu.2026.1706375)
Supplement: Supplementary file 1 [file Table1.docx]

Table S1

|  | coefficient | HR | SE | P Value |
| --- | --- | --- | --- | --- |
| Intercept | -1.599 | 0.20 | 0.501 | 0.00 |
| First-line | 0.537 | 1.71 | 0.210 | 0.01 |
| AFP>400 | 1.716 | 5.56 | 0.879 | 0.05 |
| Median follow-up | -0.008 | 0.99 | 0.013 | 0.56 |
| Locoregional Therapy | -0.373 | 0.69 | 0.275 | 0.17 |
| Main portal vein invasion | -1.099 | 0.33 | 0.720 | 0.13 |

Table S1 The summary for PFS meta-regression

Figure S1


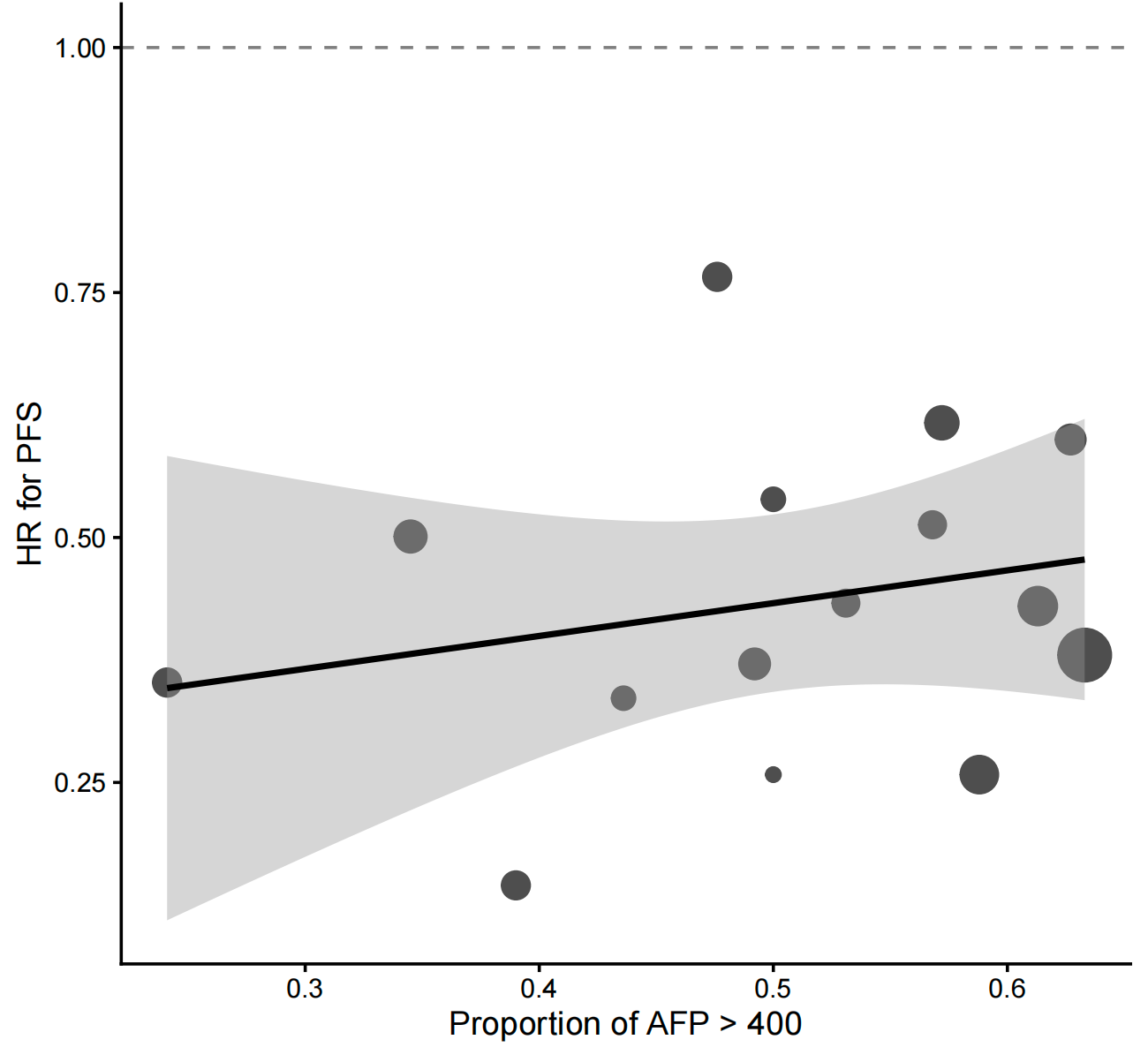


Figure S1 Meta-regression bubble plot of AFP

Figure S2


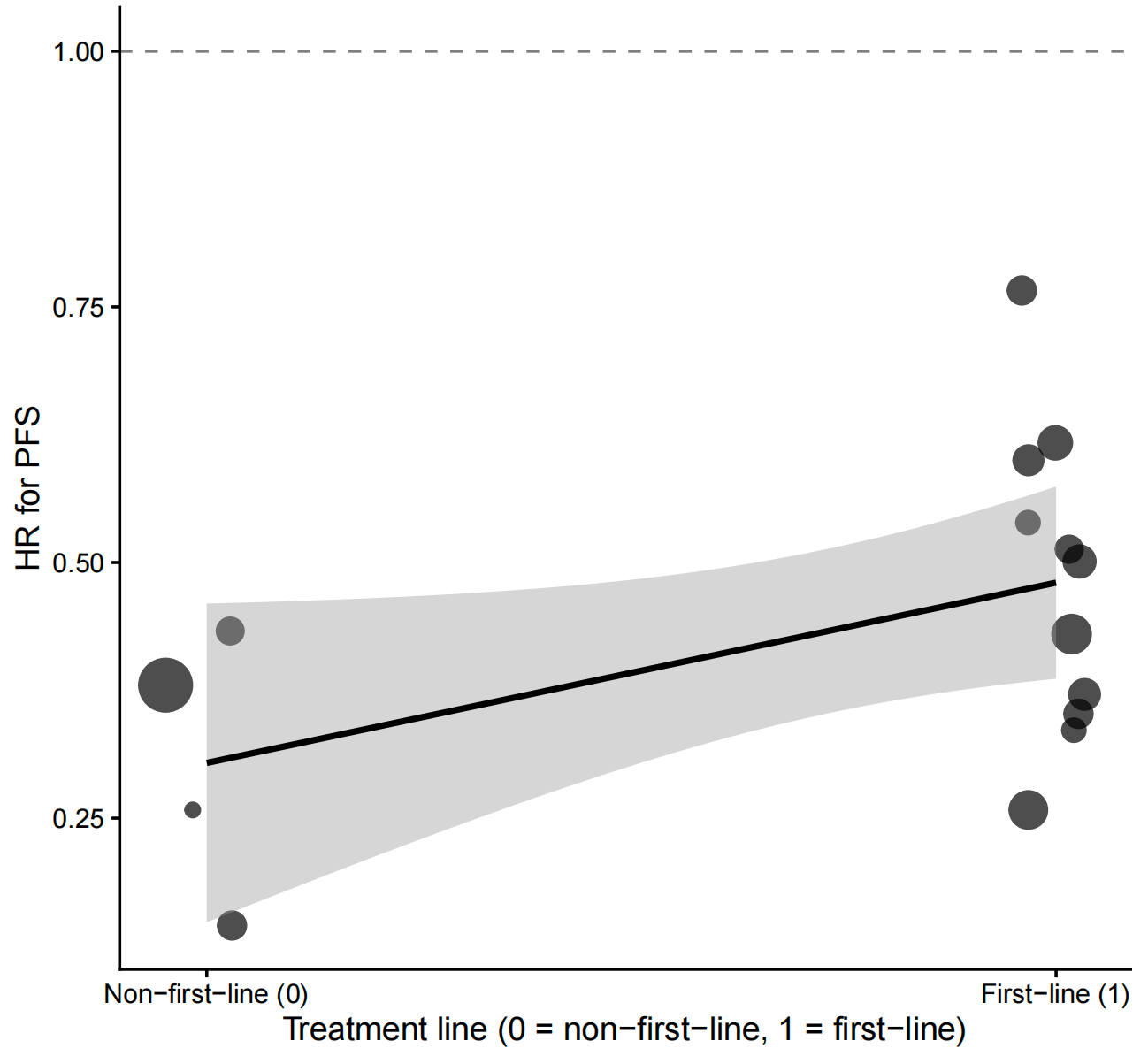


Figure S2 Meta-regression bubble plot of treatment line

Table S2

|  | coefficient | HR | SE | P Value |
| --- | --- | --- | --- | --- |
| Intercept | -0.915 | 0.40 | 0.768 | 0.23 |
| First-line | -0.159 | 0.85 | 0.337 | 0.64 |
| AFP>400 | 0.524 | 1.69 | 1.375 | 0.70 |
| Median follow-up | 0.01 | 1.01 | 0.023 | 0.67 |
| Locoregional Therapy | -0.261 | 0.77 | 0.414 | 0.53 |
| Main portal vein invasion | -1.253 | 0.29 | 1.080 | 0.25 |

Table S2 The summary for OS meta-regression
